# Impact of malocclusion on oral health-related quality of life: insights from children with and without hypodontia

**DOI:** 10.1007/s00056-025-00580-3

**Published:** 2025-03-21

**Authors:** Farnaz Duisterwinkel, Wim P. Krijnen, Bart J. Polder, Yijin Ren, Anne Marie Kuijpers-Jagtman

**Affiliations:** 1 Department of Orthodontics, University Medical Center Groningen, University of Groningen, Hanzeplein 1, 9713 GZ Groningen, The Netherlands; 2 Johan Bernoulli Institute for Mathematics and Computer Science, University of Groningen, 9700 AK Groningen, The Netherlands; 3 Center for Special Dental Care, Vogellanden, Hyacinthstraat 66A, 8013 XZ Zwolle, The Netherlands; 4 Faculty of Dentistry, Universitas Indonesia, Campus Salemba, Jalan Salemba Raya No. 4, 10430 Jakarta, Indonesia; 5 Dentofacial Orthopedics, School of Dental Medicine/Medical Faculty, University of Bern, Freiburgstrasse 7, 3010 Bern, Switzerland

**Keywords:** Tooth agenesis, Orthodontics, Anodontia, Orthodontic treatment need, Dental esthetics, Zahnagenesie, Kieferorthopödie, Anodontie, Kieferorthopädischer Behandlungsbedarf, Zahnästhetik

## Abstract

**Objective:**

To assess the impact of malocclusion on oral health-related quality of life (OHRQoL) and to compare the impact of malocclusion in children with and without hypodontia.

**Methods:**

Children aged 10–16 years with ≥ 5 missing teeth and without hypodontia completed the Malocclusion Impact Questionnaire (MIQ) to assess the impact of malocclusion on OHRQoL. The Child Perception Questionnaire 11–14 years short form (CPQ11-14-ISF16) was used to verify the validity of the MIQ. Demographic and orthodontic data were collected. Internal consistency and validity of the MIQ were analyzed. MIQ scores were compared using an independent t‑test. Regression analysis was performed to identify predictors of the MIQ score.

**Results:**

A total of 92 participants completed the MIQ, and 52 participants the CPQ11-14-ISF16. The MIQ showed excellent internal consistency (Cronbach’s α 0.89) and good criterion validity with CPQ11-14-ISF16 (r = 0.58). No significant difference in the impact of malocclusion on OHRQoL between the groups (*p* = 0.15) was found. Age (*p* < 0.05), sex (*p* < 0.001), and general appearance (*p* < 0.001) significantly predicted OHRQoL scores in the regression analysis. Multilevel analysis showed that the group and age effects were nonsignificant and that sex and general appearance were predictive for the MIQ score.

**Conclusion:**

This study suggests that the MIQ is a useful tool to assess the impact of malocclusion on OHRQoL in the orthodontic field. Differences in the impact of malocclusion on OHRQoL between children with and without hypodontia of ≥ 5 teeth were limited. It may be beneficial delaying treatment until the patient expresses a subjective treatment need which may reduce overtreatment of children with hypodontia.

## Abstract (de)

**Zielsetzung:**

Bewertung der Auswirkungen von Zahnfehlstellungen auf die mundgesundheitsbezogene Lebensqualität (OHRQoL) und Vergleich der Auswirkungen von Zahnfehlstellungen bei Kindern mit und ohne Hypodontie.

**Methoden:**

Kinder im Alter von 10-16 Jahren mit ≥ 5 fehlenden Zähnen und ohne Hypodontie füllten den Malocclusion Impact Questionnaire (MIQ) aus, um die Auswirkungen der Malokklusion auf die OHRQoL zu beurteilen. Der Child Perception Questionnaire 11-14 years short form (CPQ11-14-ISF16) wurde zur Überprüfung der Validität des MIQ verwendet. Demographische und kieferorthopädische Daten wurden erhoben. Innere Konsistenz und Validität des MIQ wurden analysiert. Die MIQ-Werte wurden mit einem unabhängigen t‑Test verglichen. Anhand einer Regressionsanalyse wurden Prädiktoren für den MIQ-Wert ermittelt.

**Ergebnisse:**

Insgesamt 92 Teilnehmende füllten den MIQ aus, 52 den CPQ11-14-ISF16. Der MIQ zeigte eine ausgezeichnete innere Konsistenz (Cronbachs α 0,89) und eine gute Kriteriumsvalidität mit dem CPQ11-14-ISF16 (r = 0,58). Es bestand kein signifikanter Unterschied zwischen den Gruppen hinsichtlich des Einflusses der Malokklusion auf die OHRQoL (*p* = 0,15). Alter (*p* < 0,05), Geschlecht (*p* < 0,001) und allgemeines Erscheinungsbild (*p* < 0,001) sagten in der Regressionsanalyse die OHRQoL-Werte signifikant voraus. Die Mehrebenenanalyse zeigte, dass die Gruppen- und Alterseffekte nicht signifikant waren und dass Geschlecht und allgemeines Erscheinungsbild prädiktiv für den MIQ-Wert waren.

**Schlussfolgerung:**

Diese Studie deutet darauf hin, dass der MIQ ein geeignetes Tool ist, um die Auswirkungen von Malokklusion auf die OHRQoL im kieferorthopädischen Bereich zu bewerten. Die Unterschiede in den Auswirkungen der Malokklusion auf die OHRQoL zwischen Kindern mit und ohne Hypodontie von ≥ 5 Zähnen waren begrenzt. Es ist möglicherweise sinnvoll, mit der Behandlung zu warten, bis der Patient einen subjektiven Behandlungsbedarf äußert, was die Überbehandlung von Kindern mit Hypodontie reduzieren könnte.

## Introduction

Hypodontia is a congenital condition characterized by the absence of one or more permanent teeth, excluding third molars. The prevalence varies by continent and gender [28], with an estimated global prevalence ranging from 2.6 to 11.3%. The incidence is also 1.37 times higher in females compared to males [11, 28]. The prevalence of hypodontia in Germany was stated between 3.5 and 6.5% [19]. The etiology of hypodontia is multifactorial and includes genetic and environmental factors [3, 32]. Tooth development is primarily regulated by genetic factors and involves complex molecular interactions [32]. Disruptions in these pathways can lead to conditions like hypodontia. Recent theories on tooth agenesis range from evolutionary changes to anatomical principles. The evolutionary changes are linked to the shortening of the maxillomandibular complex during human evolution, resulting in a corresponding decrease in the number of teeth. The anatomical principles are based on the hypothesis that certain regions of the dental lamina are more susceptible to environmental factors during tooth development [3, 11]. Patients with hypodontia may encounter problems such as reduced masticatory ability, pronunciation problems, esthetic concerns, and periodontal problems [9, 13]. Management of hypodontia differs from other medical interventions because patients are mostly young and healthy. Unlike their peers these patients and their families often make long-term commitment to complex treatment including orthodontics, prosthodontics, and oral surgery. On the other hand, patients and their families should understand the implications of treatment, preferably based on patient-centered outcome measures [2]. Unfortunately, commonly used outcome measures for treatment of hypodontia are clinician-focused and have limited value in supporting patients during decision making [4]. Oral health-related quality of life (OHRQoL) measures the impact of oral health on daily life in terms of oral symptoms, functional limitations, and emotional and social wellbeing [22]. Several instruments have been developed to measure OHRQoL in children, but most of these focus on overall oral health rather than specific dental issues [13]. Hence, there is a growing demand for condition-specific questionnaires. A hypodontia-specific questionnaire was applied in Dutch children with and without hypodontia [13]. However, the groups were difficult to compare because of the small sample size. Also, when the questions for the affected group were applied to the unaffected group, half of the questions could not be answered.

The impact of malocclusion is usually not taken into consideration when assessing OHRQoL. A frequently used instrument is the more general CPQ11-14 questionnaire. In the study of Bhatia et al. [8], malocclusion had a significant impact on the emotional, social, and functional well-being, while problems such as dental caries and periodontal disease were less important. It can be assumed that a questionnaire developed specifically for malocclusion will provide advantages of having a limited number of questions, being less time-consuming, and providing better understanding of the patients’ concerns [8]. Thereto, the Malocclusion Impact Questionnaire (MIQ) was developed for children aged 10–16 years with malocclusion [7, 27].

Assessment of OHRQoL in children with hypodontia is valuable in clinical practice and decision-making as malocclusion severity may affect their quality of life and contribute to psychological stress and anxiety [18]. Several studies have examined OHRQoL in children aged 11–17 with and without hypodontia, but findings have been inconsistent [13, 21, 23, 33]. Some studies found worse OHRQoL in children with hypodontia compared to nonaffected children [23, 33], while others found no difference in psychosocial status between affected and nonaffected individuals with a comparable treatment need [13, 21]. Furthermore, the use of different questionnaires has contributed to heterogeneity of data and difficulty in making conclusive statements. As a result, there is insufficient comprehensive research available on how malocclusion and hypodontia affect the OHRQoL in children. To address this gap, this study aimed to assess the impact of malocclusion on OHRQoL and to compare the perceived impact of malocclusion on the lives of children, aged 10–16, with hypodontia to that of peers of the same age who are in need for regular orthodontic treatment.

## Materials and methods

This study was approved by the Medical Ethics Review Board of the University Medical Center Groningen (METc UMCG), Groningen, The Netherlands (METc 2020/431). All parents/caregivers and participants gave written informed consent before participating in the study. This cross-sectional study included 92 children aged 10–16 years. Participants were recruited from two centers for special dental care in Groningen and Zwolle, the Netherlands and allocated to a control group (C) and a hypodontia group (H). Children in (C) were patients in need of regular orthodontic treatment in the same age range. Children in (H) were patients with hypodontia missing 5 or more teeth, excluding third molars, who were referred to the two centers. A total of 26 patients of the (H) group had undergone interceptive orthodontic treatment, some of them in combination with preorthodontic prosthetic treatment. (C) children had to be at the start of their orthodontic treatment before orthodontic appliances were placed. Exclusion criteria for both groups were severe skeletal discrepancy, complex medical history or learning disability that would impair understanding the questionnaire, cleft lip and palate and craniofacial syndromes and patients with history of previous orthodontic treatment (applicable only for (C)). The sample size calculation using G*power 3.1 [12] indicated that 29 participants per group were necessary to detect a minimum effect size of 0.8 for differences in quality of life (QoL) scores between individuals with and without tooth agenesis. The rationale to set the effect size was based on earlier, similar studies [20, 21]. A medium effect size is defined as 0.5, while a ‘large effect size’ is defined as 0.8 [10]. Therefore, the effect size was set at 0.8 with 80% power at a 5% level of significance. To account for potential incomplete data from questionnaires, recruitment continued until each group included at least 46 participants.

## Questionnaires

The Malocclusion Impact Questionnaire (MIQ) was used to measure the impact of the malocclusion on OHRQoL [6]. The Dutch version of the MIQ was developed earlier according to the translation and cross-cultural adaptation guideline by Beaton et al. (2000) which comprises five stages [4, 11]. Stages I–III describe the translation process, consisting of forward–backward translations. In stage IV, an expert committee and the translators discuss wording options and other lacunes in the questionnaire until consensus is reached. In the final stage, the translated version is tested to check the content validity among the targeted population [14]. Since it was the first time to use the final questionnaire in a Dutch population, the ‘Child Perception questionnaire 11–14 years short form’ (CPQ11-14-ISF16) was additionally used to check construct validity [15, 16]. The latter questionnaire was designed for children aged 11–14; therefore, participants younger than 11 years were excluded from answering the CPQ11-14-ISF16. Children aged 15 and 16 were included because comprehension of the questionnaire’s components would not be compromised [21].

Participants were either given a paper and pen version of the questionnaire to complete in the hospital’s waiting room or an e‑mail link to the survey was sent to their parents/caregivers. For the electronic questionnaires, RedCap 10.0.23 (© 2022 Vanderbilt University, Nashville, TN, USA) was used.

## Variables and their measurements

The MIQ consists of 2 global and 17 more specific questions which can be divided into four domains, ‘Domain 1: How the way your teeth look might make you feel’, ‘Domain 2: How the way your teeth might affect you’, ‘Domain 3: The way your teeth look makes you worried or concerned’, ‘Domain 4: Other ways your teeth might affect you’. The two global questions were scored with a 5-point severity scale, from 0 = ‘Not at all’ to 4 = ‘Very much’. The 17 items have a 3-point response format. Negatively worded questions (‘Nervous’, ‘Shy’) are scored as 0 don’t/doesn’t, 1 = a bit, 2 = very/a lot. Positively worded questions (‘happy’, ‘good looking’, ‘confident’) are scored as 0 = very/a lot, 1 = a bit, 2 = don’t. Per participant the sum score of the 17 items was taken as the total MIQ, resulting in a minimum of 0 and maximum of 34.

The CPQ11-14-ISF16 consists of 16 items divided into four domains ‘oral symptoms’, ‘functional limitations’, ‘emotional well-being’ and ‘social well-being’ [15, 16]. The items are scored with a 5-point severity scale, from 0 = ‘never’ to 4 = ‘every day/almost every day’. The minimum score was 0 and the maximum 64. For both questionnaires higher scores indicate poorer OHRQoL.

Demographic and orthodontic data for each patient were collected from the computer-based patient records by the primary investigator including sex, age, satisfaction on general appearance and Angle classification. Additionally, in the (H) group the following data were collected: microdontia or having > 1 abnormally small/peg-shaped teeth (yes/no), number of missing teeth, interceptive orthodontic treatment (yes/no).

All data was collected in Microsoft Excel (Microsoft Corp, Redmond, WA, USA) and statistically analyzed by the programming language R [29].

## Statistical evaluation

To determine the validity of the Dutch version of the MIQ, the Pearson’s product–moment correlation between the MIQ and the CPQ11-14-ISF16 was assessed with its 95% confidence interval (95% CI). Values between 0.50 and 0.75 mean a moderate to good correlation and those greater than 0.75 indicate very good to excellent correlation [31]. Cronbach’s α was calculated to determine the internal consistency and reliability with the minimum acceptable value set at 0.70 [17].

The internal properties of the questionnaires were analyzed to assess the quality of each questionnaire item. For this purpose, using the kernel smoothing method, item characteristic curves (ICC) and point polyserial correlations were computed and analyzed [25]. This method was used to estimate the latent variable that estimates the ICC. Note that the loadings are similar to loadings of a confirmatory factor analysis. Correlations ≤ 0.39 indicate low quality of a questionnaire item [14, 28].

For the analysis of the main hypothesis an independent t‑test was performed. Item impact scores for each MIQ item were determined for both groups [7]. These were calculated by multiplying the proportion of participants indicating a moderate or significant impact score (scores 1 or 2) with the mean sample score for that question. To investigate predictors of the MIQ score, regression analyses with a single outcome (univariate analysis) and several covariates as independent variables (multilevel analysis) were performed.

## Results

A summary of demographic and orthodontic data for the two groups is shown in Table 1. Children younger than 11 years were excluded from the CPQ11-14-ISF16 questionnaire. Also, there was a group of children who did not complete the CPQ11-14-ISF16 for various reasons and were therefore omitted, leading to a difference in sample size between the two questionnaires.

**Table 1:** Descriptive characteristics of the control and hypodontia samples, divided according to the completed questionnaires (Malocclusion Impact Questionnaire [MIQ] or Child Perception Questionnaire 11–14 years short form [CPQ11-14-ISF16]) Deskriptive Merkmale der Kontroll- und der Hypodontie-Studiengruppe, aufgeteilt nach den ausgefüllten Fragebögen (Malocclusion Impact Questionnaire [MIQ] bzw. Child Perception Questionnaire 11-14 years short form [CPQ11-14-ISF16])

	MIQ		CPQ11-14-ISF16	
	Control *N* (%)	Hypodontia *N* (%)	Control *N* (%)	Hypodontia *N* (%)
*Demographics*				
Age				
10	9 (20%)	10 (22%)	n. a.^a^	n. a.^a^
11–16	37 (80%)	36 (78%)	30 (100%)	20 (100%)
Sex				
Male	24 (52%)	21 (46%)	14 (44%)	10 (50%)
Female	22 (48%)	25 (54%)	18 (56%)	10 (50%)
General appearance				
Very happy	1 (2%)	1 (2%)	6 (19%)	2 (10%)
Quite happy	4 (9%)	8 (17%)	24 (75%)	13 (65%)
A little happy	28 (61%)	27 (59%)	1 (3%)	4 (20%)
Not very happy	13 (28%)	10 (22%)	1 (3%)	1 (5%)
Not at all happy	0 (0%)	0 (0%)	0 (0%)	0 (0%)
*Orthodontic data*				
Angle classification				
Class I	13 (28%)	17 (37%)	10 (31%)	7 (35%)
Class II/I	17 (37%)	13 (28%)	11 (34%)	5 (25%)
Class II/2	13 (28%)	13 (28%)	9 (28%)	7 (35%)
Class III	2 (4%)	3 (7%)	2 (6%)	1 (5%)
Number of missing teeth				
0	46 (100%)	0 (0%)	32 (100%)	0 (0%)
1–4	0 (0%)	0 (0%)	0 (0%)	0 (0%)
5	0 (0%)	8 (17%)	0 (0%)	6 (19%)
6	0 (0%)	7 (15%)	0 (0%)	2 (6%)
7	0 (0%)	6 (13%)	0 (0%)	4 (13%)
8	0 (0%)	3 (7%)	0 (0%)	2 (6%)
9	0 (0%)	4 (9%)	0 (0%)	1 (3%)
> 10	0 (0%)	18 (39%)	0 (0%)	17 (53%)
Microdontia or > 1 abnormally small/peg-shaped teeth				
Yes	0 (0%)	25 (54%)	0 (0%)	12 (60%)
No	46 (100%)	21 (46%)	32 (100%)	8 (40%)
Interceptive treatment				
Yes	0	26 (28%)	0	14
No	46 (100%)	65 (72%)	32 (100%)	6

^a^ children < 11 years were excluded from the CPQ11-14-ISF16

## Quality of the questionnaires

### Validity and internal consistency

The correlation between overall scores of CPQ11-14-ISF16 and MIQ was r = 0.58 (95% confidence interval [CI] 0.36–0.74; *p* < 0.001) showing that the MIQ has good criterion validity with a commonly used measure of OHRQoL. Table 2 shows that Cronbach’s α was high (0.89) for the MIQ (total). Table 3 shows an acceptable Cronbach’s α (0.79) for the CPQ (total). Removing questionnaire item 19 would give an excellent Cronbach’s α for the MIQ (Table 2). Removing items 2, 7, 14, and 16 would improve the α of the CPQ (total) (Table 3).

**Table 2:** Prevalence of the answer possibilities on the Malocclusion Impact Questionnaire (MIQ) items; Cronbach’s α and polyserial correlations coefficients Prävalenz der Antwortmöglichkeiten auf die Items des Malocclusion Impact Questionnaire (MIQ); Cronbachs α und polyserielle Korrelationskoeffizienten

	Questionnaire item		Likert scale 0–4					α if item deleted	Point polyserial correlation coefficient
			0 (in %)	1 (in %)	2 (in %)	3 (in %)	4 (in %)		
*Global questions (2)*^*a*^	MIQ_1	Overall, how much do your teeth bother you?	31	34	21	12	2	0.89	0.74
	MIQ_2	Overall, how much do your teeth affect your life?	39	39	16	6	–	0.89	0.65
*Specific questions (17)*^*b*^	MIQ_3	Happy	39	51	10	–		0.88	0.74
Domain 1: How the way your teeth look might make you feel	MIQ_4	Good looking	26	58	16			0.89	0.56
	MIQ_5	Confident	47	44	9			0.88	0.71
	MIQ_6	Normal	82	18	–			0.89	0.58
	MIQ_7	Sad	84	15	1			0.89	0.54
	MIQ_8	Nervous	78	20	2			0.89	0.51
	MIQ_9	Shy	77	21	2			0.89	0.60
Domain 2: How the way your teeth look might affect you	MIQ_10	Smile	60	30	10			0.89	0.63
	MIQ_11	Laugh	64	27	9			0.88	0.76
	MIQ_12	Seeing photographs	70	27	3			0.88	0.70
	MIQ_13	Talk in public	82	18	–			0.89	0.58
Domain 3: The way your teeth look makes you worried or concerned	MIQ_14	Others nicer teeth	61	30	9			0.89	0.60
	MIQ_15	Being bullied	90	9	1			0.89	0.44
	MIQ_16	Making friends	95	5	–			0.89	0.54
	MIQ_17	Fitting in with friends	95	4	1			0.89	0.52
Domain 4: Other ways your teeth might affect you	MIQ_18	Cover with hand	76	20	4			0.89	0.66
	MIQ_19	Biting some foods	72	25	3			0.90	0.35
*α MIQ (total)* *=* *0.89*	–								

^a^ 5-point response format from 0 = Not at all, 1 = A little, 2 = Somewhat, 3 = Quite a bit, 4 = Very much

^b^ 3-point response format: Negatively worded questions (Nervous, Shy): 0 = don’t/doesn’t, 1 = a bit, 2 = very/a lot. Positively worded questions happy, good looking, confident: 0 = very/a lot, 1 = a bit, 2 = don’t

**Table 3:** Prevalence of the answer possibilities on the Child Perception Questionnaire 11–14 years short form (CPQ11-14-ISF16) items; Cronbach’s α and point polyserial correlation coefficients Prävalenz der Antwortmöglichkeiten auf die Items des Child Perception Questionnaire 11-14 Jahre Kurzform (CPQ11-14-ISF16); Cronbachs α und polyserielle Punktkorrelationskoeffizienten

	Questionnaire items		Likert scale 0–4^a^					α if item deleted	Point polyserial correlation coefficient
			0 (in %)	1 (in %)	2 (in %)	3 (in %)	4 (in %)		
Oral symptoms	CPQ_1	Pain in teeth	86	10	2	2	–	0.78	0.60
	CPQ_2	Sores in mouth	67	23	10	–	–	0.81	0.35
	CPQ_3	Bad breath	74	22	4	–	–	0.79	0.48
	CPQ_4	Food stuck	35	49	12	4	–	0.76	0.75
Functional limitations	CPQ_5	Longer eating	86	8	6	–	–	0.79	0.56
	CPQ_6	Difficulty biting/chewing	92	6	2	–	–	0.79	0.42
	CPQ_7	Difficulty words	94	6	–	–	–	0.80	0.10
	CPQ_8	Difficulty drinking	78	20	2	–	–	0.79	0.46
Emotional well-being	CPQ_9	Felt irritable/frustrated	76	18	6	–	–	0.76	0.75
	CPQ_10	Felt shy/embarrassed	80	16	2	2	–	0.76	0.76
	CPQ_11	Being concerned other people think	84	16	–	–	–	0.79	0.41
	CPQ_12	Been upset	90	10	–	–	–	0.79	0.39
Social well-being	CPQ_13	Avoided smiling/laughing	82	12	4	2	–	0.76	0.77
	CPQ_14	Argues with others/family	98	2	–	–	–	0.80	0.01
	CPQ_15	Teased	100	–	–	–	–	–	–
	CPQ_16	Asked questions	82	16	2	–	–	0.80	0.35
*α CPQ (total)* *=* *0.79*	–								

^a^ 5-point severity scale, from 0 = never to 4 = everyday/almost everyday

### Item analysis

**Fig. 1:**
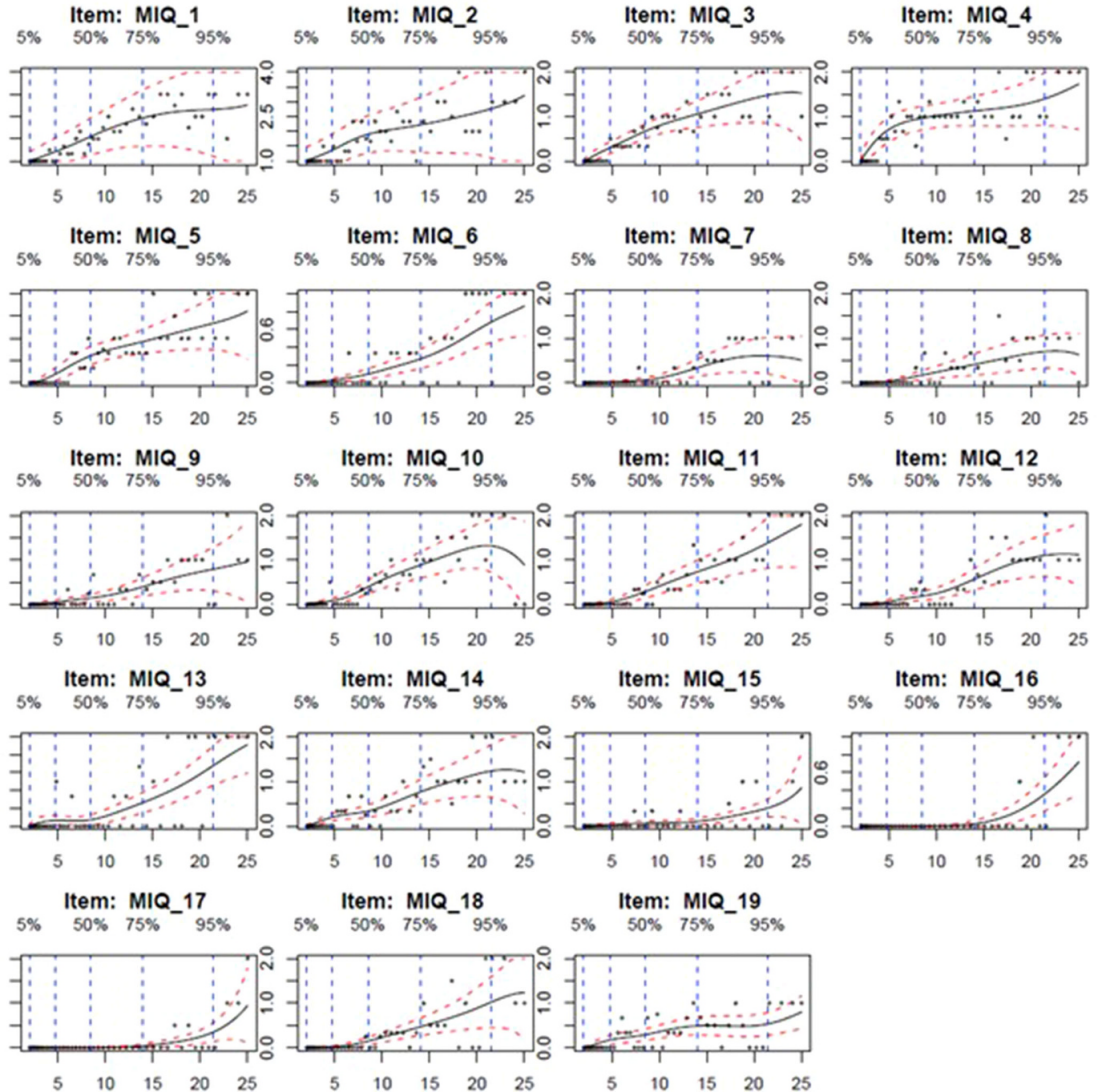
Item characteristic curves of the Malocclusion Impact Questionnaire (MIQ) using kernel smoothing method; X‑axis: questionnaire scores, Y‑axis: scores on Likert scale Item-Charakteristik-Kurven des Malocclusion Impact Questionnaire (MIQ) unter Verwendung der Kernel-Glättungsmethode; X‑Achse: Fragebogen-Scores, Y‑Achse: Scores auf der Likert-Skala

**Fig. 2:**
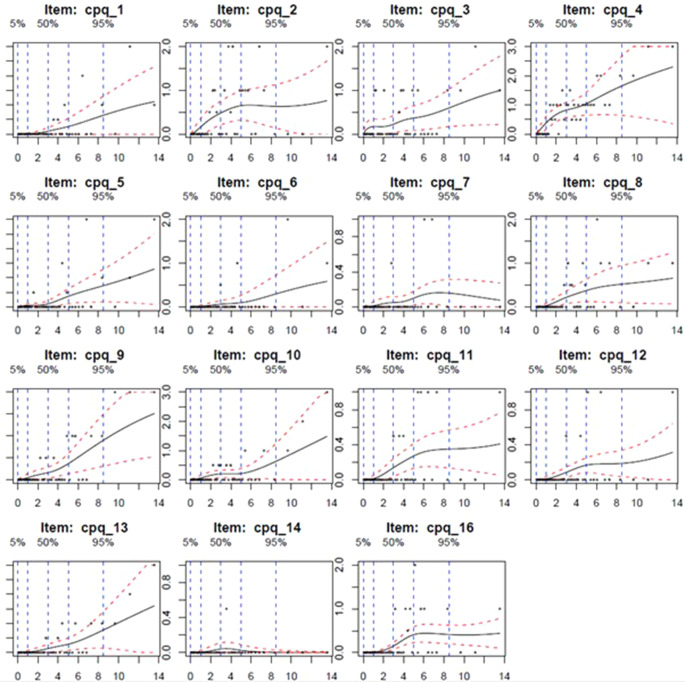
Item characteristic curves of the Child Perception Questionnaire 11–14 years short form (CPQ) using kernel smoothing method; X‑axis: questionnaire scores, Y‑axis: scores on Likert scale Item-Charakteristik-Kurven des Child Perception Questionnaire 11-14 Jahre Kurzform (CPQ) unter Verwendung der Kernel-Glättungsmethode; X‑Achse: Fragebogenwerte, Y‑Achse: Werte auf der Likert-Skala

Point polyserial correlations (Table 2) for the MIQ showed that mainly MIQ_19 had low correlation to the total questionnaire score. For the CPQ11-14-ISF16 (Table 3), it seems that items 2, 7, 14, 15, and 16 were questions that elicited poor response patterns. These findings were also illustrated with the item characteristic curves for both questionnaires (Figs. 1 and 2). The course of the curve is an indicator for the quality of the question. Generally, higher questionnaire scores give higher scores on the Likert scale. For example, MIQ_4 was a valuable question because it was sensitive for low MIQ scores, while MIQ_8 was not because even with a high MIQ score the chance of scoring low on the Likert scale was large.

## Descriptive analysis

Table 4 shows the MIQ total scores for the control and hypodontia group. The hypodontia group had higher total MIQ scores (mean 6.6, standard deviation [SD] 5.3) than the control group (mean 5.1, SD 5.1), but the independent t‑test showed that the difference was not statistically significant (*p* = 0.15).

**Table 4:** Responses Malocclusion Impact Questionnaire (MIQ) for the control (*n* = 46) and hypodontia group (*n* = 46) Antworten Malocclusion Impact Questionnaire (MIQ) für die Kontrollgruppe (*n* = 46) und die Hypodontiegruppe (*n* = 46)

MIQ total score^a^	*N*	Median	Mean	SD	Min	Max	95% CI	*P*
Control	46	4	5.1	5.1	0	22	[−0.22, 0.03]	0.15
Hypodontia	46	5	6.6	5.3	0	20		

^a^ MIQ total score = MIQ3 to MIQ19

*SD* standard deviation, *Min* minimum, *Max* Maximum, *95% CI* 95% confidence interval

An overview of the item impact scores is shown in Table 5. A higher score indicates greater significance of the question within the experimental group, e.g., the hypodontia group exhibited the highest score (0.75) for MIQ_4. This question concerning ‘How the way your teeth look might make you feel’, appears to have a greater impact in the hypodontia group as opposed to the control group.

**Table 5:** Item impact scores for Malocclusion Impact Questionnaire (MIQ) items Item-Impact-Scores für Items des Malocclusion Impact Questionnaire (MIQ)

Question		Control	Hypodontia
*Domain 1: How the way your teeth look might make you feel*			
MIQ_3	Happy	0.42	0.44
MIQ_4	Good looking	0.59	0.75
MIQ_5	Confident	0.24	0.44
MIQ_6	Normal	0.02	0.05
MIQ_7	Sad	0.02	0.04
MIQ_8	Nervous	0.24	0.06
MIQ_9	Shy	0.00	0.06
*Domain 2: How the way your teeth look might affect you*			
MIQ_10	Smile	0.39	0.28
MIQ_11	Laugh	0.35	0.22
MIQ_12	Seeing photographs	0.28	0.14
MIQ_13	Talk in public	0.05	0.02
*Domain 3: The way your teeth look makes you worried or concerned*			
MIQ_14	Others nicer teeth	0.24	0.29
MIQ_15	Being bullied	0.01	0.01
MIQ_16	Making friends	0.00	0.00
MIQ_17	Fitting in with friends	0.00	0.01
*Domain 4: Other ways your teeth might affect you*			
MIQ_18	Cover with hand	0.28	0.07
MIQ_19	Biting some foods	0.13	0.20

## Predictors of OHRQoL

Age (*p* < 0.05), sex (*p* < 0.001), and general appearance (*p* < 0.001) significantly predicted OHRQoL scores in the univariate regression for both groups (Table 6). Multilevel analysis showed that group and age effect were nonsignificant and that sex and general appearance were predictive for the MIQ score. The model explains the variance of the MIQ score for 44% (R^2^ = 0.438).

**Table 6:** Regression analysis procedure to investigate predictors for the Malocclusion Impact Questionnaire (MIQ) score for the control and hypodontia groups Regressionsanalyseverfahren zur Untersuchung der Prädiktoren für den Malocclusion Impact Questionnaire (MIQ) für die Kontroll- und die Hypodontiegruppe

Variables	Univariate regression analysis		Multilevel analysis	
	(Estimated) effect	*P*-value	(Estimated) effect	*P*-value
Group H^a^	1.847	0.103	0.694	0.436
Age	0.792	*0.012*	0.452	0.088
Sex B^b^	4.316	*<* *0.001*	2.913	*0.002*
General appearance	−4.749	*<* *0.001*	−3.837	*<* *0.001*
Angle classification	1.016	0.688	2.237	0.351
Adjusted R	–		0.438	

^a^ hypodontia (H) group

^b^ male (A) and female (B)

## Discussion

This study examined the impact of malocclusion on OHRQoL in children with and without hypodontia, using the MIQ. We used the CPQ11-14-ISF16 for comparison because it is a generic measure of OHRQoL [16], which is well-known and often used in dentistry, and it has been used also in earlier cross-validation studies [6, 7]. The results suggest that there is a limited impact of malocclusion on OHRQoL between children with hypodontia of ≥ 5 teeth and children in need for regular orthodontic treatment. Currently there is no gold standard measure for assessing OHRQoL in children, particularly in the context of orthodontics. While the CPQ is useful for comparing different populations, it has limited ability to assess the effects of specific conditions such as hypodontia. The MIQ questionnaire demonstrated better reliability and quality compared to the CPQ11-14-ISF16. These findings are consistent with another study that explored face and content validity of the CPQ11-14-ISF16, indicating that a condition specific questionnaire, like the MIQ, is preferable when conducting such a study [7, 24].

Our study identified age, sex, and general appearance as significant predictors of OHRQoL. Females experienced a greater negative impact on OHRQoL, which worsened with age. This may be due to increased awareness of appearance during early adolescence, leading to more concerns about the teeth [13, 26]. These findings align with a recent Dutch study on children with severe hypodontia, which found that older children and females scored lower on OHRQoL, particularly in areas such as facial appearance, smile, jaw appearance, social life, and emotional well-being [1]. In contrast, other studies did not show significant correlations between OHRQoL and factors such as age, sex, and psychosocial status in children with and without hypodontia [21, 30]. Laing et al. [21] reported that functional complaints were linked to the severity of the condition and the number of missing teeth, with some individuals not experiencing issues because retained primary teeth masked the problem. Raziee et al. [30] found a significant correlation between OHRQoL and the number of site-specific missing teeth, though their inclusion of young adults (up to 18 years) may explain this difference. Filius et al. [13] found no sex differences in OHRQoL. These discrepancies might be due to differences in OHRQoL measures, study location, control group characteristics, and age range. Additionally, in the Netherlands, patients with severe hypodontia commonly undergo interceptive orthodontic and restorative interventions, usually initiated around age 9–10. In our cohort, 28% of children in the hypodontia group received interceptive treatment. Considering the study’s age range of 10–16 years, this early intervention may explain the limited differences found between the control and hypodontia group. Other factors that may explain the limited differences between the hypodontia and control group could be that routine orthodontic patients were similarly affected by psychosocial concerns related to their teeth, although for different reasons. In this study, 65% of the children needing regular orthodontic treatment had an Angle class II malocclusion. In both groups, most participants rated their satisfaction with their general appearance as ‘a little happy’ or ‘not very happy’, suggesting that they were equally dissatisfied with their looks. Also, some subjects in the hypodontia group may not have been affected by visible gaps in their dentition as they were unaware they had missing permanent teeth [21].

Upon closer examination of the item impact scores, it was observed that all scores in Domain 1, except for MIQ_8, were higher in the hypodontia group. This suggests that children with hypodontia are more conscious and insecure about their dental appearance than children without hypodontia. However, they do not seem to be socially affected by that as much as the latter group. Items such as speaking and laughing in public did not have a significant psychosocial impact on children with hypodontia, which is consistent with earlier findings of Filius et al. [13]. Item MIQ_19 which pertains to biting some foods scored slightly higher in the hypodontia group. This limited effect may be due to age, as most of the children in the study still had their deciduous teeth. Chewing difficulties were only observed in patients who had lost their deciduous teeth without successors as noted in previous studies [1, 18].

Overall, the results of this study for the item impact scores were comparable with the New Zealand sample, but less so with the UK sample from the 2019 study by Benson et al. [7]. Notably, the UK sample was recruited from an urban area, while the participants in the present study were recruited from a more rural region of the Netherlands.

Previous studies have reported severe functional and psychosocial problems in patients with hypodontia [23, 33] which were not confirmed in the present study. This could be due to various factors such as cultural differences, absence of a control group, and the use of a different OHRQoL measure. Differences in national health insurance systems may also play a role, as most patients with hypodontia in the Netherlands receive treatment at specialized dental care centers and costs are covered by the national health system [13].

### Clinical relevance

The findings of this study suggest that the MIQ is a valuable tool for assessing OHRQoL in future orthodontic research. There were only minor differences in the impact of malocclusion on OHRQoL between children with and without hypodontia. Given the limitations of this study, these results should be interpreted with caution. Though it may be beneficial delaying treatment until the point where patients’ and caregivers’ treatment needs converge, which could help ease the burden of care for children with hypodontia. The MIQ could also be useful in gaining a better understanding of patients’ concerns before orthodontic treatment begins by administering it before the initial consultation. This would allow clinicians to address specific patient concerns already during their first visit.

### Limitations

This study has some limitations including its cross-sectional design and the potential influence of parental assistance in completing the digital questionnaires. It is important to note that this study was conducted in the northern region of the Netherlands and results may not be generalizable to other regions or countries. Furthermore, caution should be exercised when interpreting the results regarding the performance of the MIQ in comparison to the CPQ11-14-ISF16, due to differences in sample size.

## Conclusion

This study suggests that the Malocclusion Impact Questionnaire (MIQ) is a useful tool to assess impact of malocclusion on oral health-related quality of life (OHRQoL) in the orthodontic field. Differences in impact of malocclusion on OHRQoL between children with and without hypodontia of ≥ 5 teeth were limited. It may be beneficial delaying treatment until the patient expresses a subjective treatment need which may reduce overtreatment for children with hypodontia.

## Data Availability

The research data are securely stored in the secured hospital’s database. Therefore, the data that support the findings of this study are available from the corresponding author upon reasonable request.
